# Biliary Breach: A Rare Case of a Biliary Pleural Fistula Without a History of Trauma

**DOI:** 10.7759/cureus.81286

**Published:** 2025-03-27

**Authors:** Andrew Liepshutz, Joshua D Batista, Brooke Merdjane, Nima Khosravani, Soni Chousleb

**Affiliations:** 1 Medicine, Florida International University, Herbert Wertheim College of Medicine, Miami, USA; 2 Medicine and Surgery, University of North Carolina at Chapel Hill, Chapel Hill, USA; 3 General Surgery, Baptist Health South Florida, Miami, USA; 4 Surgery, Baptist Health South Florida, Miami, USA

**Keywords:** biliary pleural fistula, bilopleural fistula, cholecystitis, cholecystopleural fistula, empyema

## Abstract

Biliary pleural fistulas (BPFs) are pathologic connections between the biliary tree and pleural cavity, allowing for abnormal flow of bile into the pleural space. This finding is rare and typically results from surgical procedures, such as chest tube placement, or trauma. We present the case of an 84-year-old male who initially presented with respiratory and gastrointestinal symptoms. Non-contrast CT of the chest, abdomen, and pelvis demonstrated a large right-sided empyema, which appeared to be continuous with a fluid-containing structure in the gallbladder fossa. Subsequent diagnostic workup revealed a biliary pleural fistula, which was ultimately treated with robotic cholecystectomy and fistula takedown. BPFs are rare entities; however, clinicians should consider them in the differential diagnoses of patients who present with both gastrointestinal and respiratory symptoms.

## Introduction

A biliopleural fistula, or biliary pleural fistula (BPF), is an abnormal connection between the biliary tree and the pleural cavity [1-7]. The term “cholecystopleural fistula” is used when the connection specifically involves the gallbladder and pleura. Fistula formation allows for the pathological flow of bile into the pleura, which is highly irritative. Patients may present with severe respiratory symptoms in addition to usual gallbladder-related symptoms, including right upper quadrant pain, diarrhea, nausea, vomiting, and food intolerance [1,2,4]. BPFs are rare entities and have been shown to occur primarily due to trauma or post-surgical procedures such as chest tube placement or percutaneous drains [1]. They may also result from liver abscesses, calculi of the intrahepatic or common bile ducts, radiofrequency ablations, or prior surgical procedures such as pneumonectomy or hepatectomy [1-7]. Given the rarity and chance of misdiagnosis of biliary pleural fistulas, they must be detected early and adequately treated to prevent severe complications and even death [1,3]. BPFs are of low frequency, and most of the literature focuses on those caused by trauma and surgical/interventional procedures [5,6]. There is limited focus on the diagnosis, treatment, and surgical techniques used. Clinicians should consider BPFs in their differential diagnosis for patients presenting with symptoms of gallbladder disease, including right upper quadrant pain, nausea, vomiting, and jaundice, with concomitant respiratory symptoms such as shortness of breath and chest pain. Given the rarity of BPFs, it is important for clinicians to understand the diagnostic process and how to treat them.

## Case presentation

An 84-year-old Hispanic male presented to the emergency department with ongoing abdominal discomfort, diarrhea, shortness of breath, and weight loss. He stated that his symptoms had been persistent for approximately eight months at the time of presentation. His past medical history includes type II diabetes mellitus, for which he was taking metformin 1000 mg daily, and glaucoma, for which he used netarsudil/latanoprost and brimonidine-timolol ophthalmic solutions. He denied any past surgical history. He also denied any previous trauma. The patient is a nonsmoker and endorses social alcohol use.

The patient was seen by an outside institution the night prior and was admitted. At that time, he underwent CT with contrast with findings concerning for empyema, as well as fluid tracking along the right mediastinum and a subphrenic collection measuring 3.6 cm. He was also noted to have leukocytosis with left shift (9% bands), lactic acidosis, hyperglycemia, and an elevated anion gap of 21. He reported that he was given unknown antibiotics during that visit.

On presentation to our institution, the patient was found to be afebrile with a heart rate of 90 beats per minute and a blood pressure of 96/56 mmHg. His respiratory rate was increased at 24 breaths per minute, and his oxygen saturation was 98% on room air. Physical examination was largely normal, with no significant cardiovascular or gastrointestinal findings. Per respiratory examination, his breathing was non-labored, although he had diminished breath sounds along the right lung base. Laboratory evaluation revealed a white blood cell count of 31.7 x 109/L with 90.7% neutrophils, 1.9% immature granulocytes, and 28.8% absolute neutrophils. His glucose was substantially elevated at 400 mg/dL and was, therefore, given 10 units/mL of insulin intravenously. His previous anion gap had resolved with the administration of IV fluids. Blood cultures were obtained, and he was started on piperacillin/tazobactam empirically.

**Figure 1:**
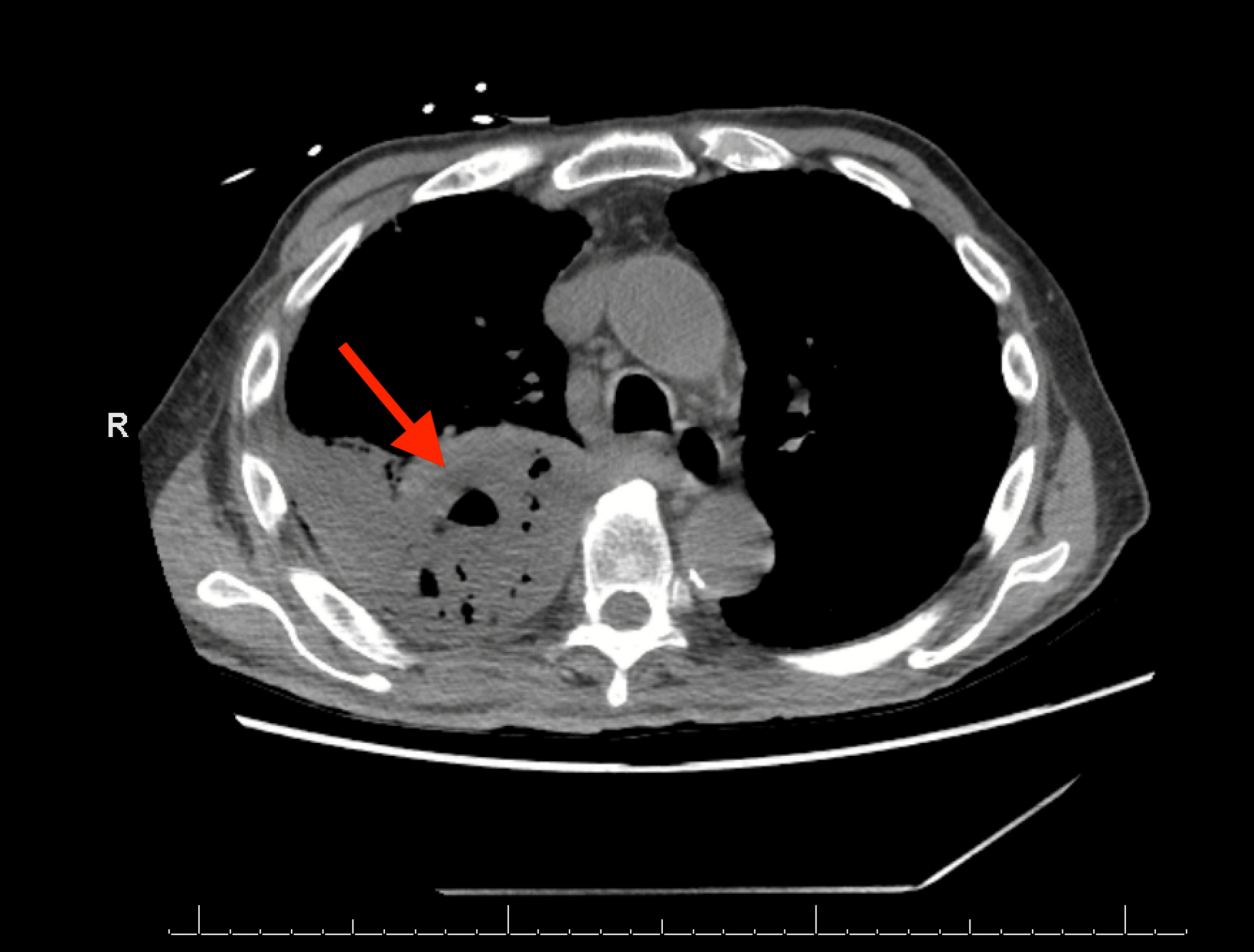
Chest CT without contrast demonstrating a right-sided pleural solid and a cystic gas lesion

**Figure 2:**
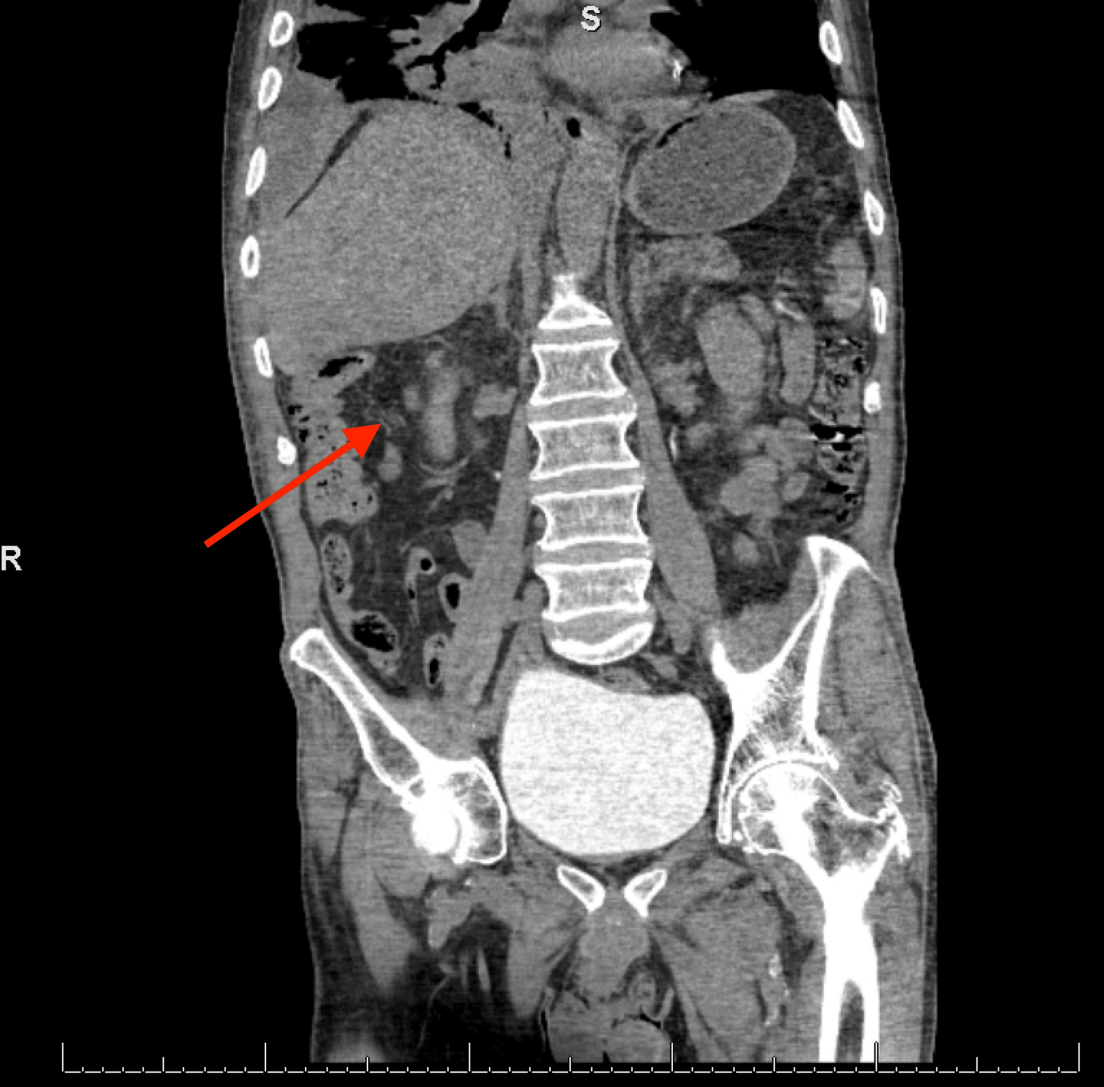
CT of the abdomen and pelvis showing a fluid-filled structure within the area of the gallbladder fossa

Initial chest X-ray showed extensive right lung opacity with the suggestion of a small right pleural effusion. Further evaluation with CT of the chest without contrast (Figure 1) demonstrated a loculated, right-sided, thick-walled pleural solid and a cystic gas lesion measuring 13.9 x 7.1 x 19.1 cm. CT abdomen and pelvis without contrast (Figure 2) revealed a tubular, fluid-containing structure (6.5 x 4.1 cm) in the area of the gallbladder fossa, raising concern for perforated cholecystitis. Notably, the perforation seemed to extend to the right hemipelvis, indicating potential continuity with the patient’s right-sided empyema.

**Figure 3:**
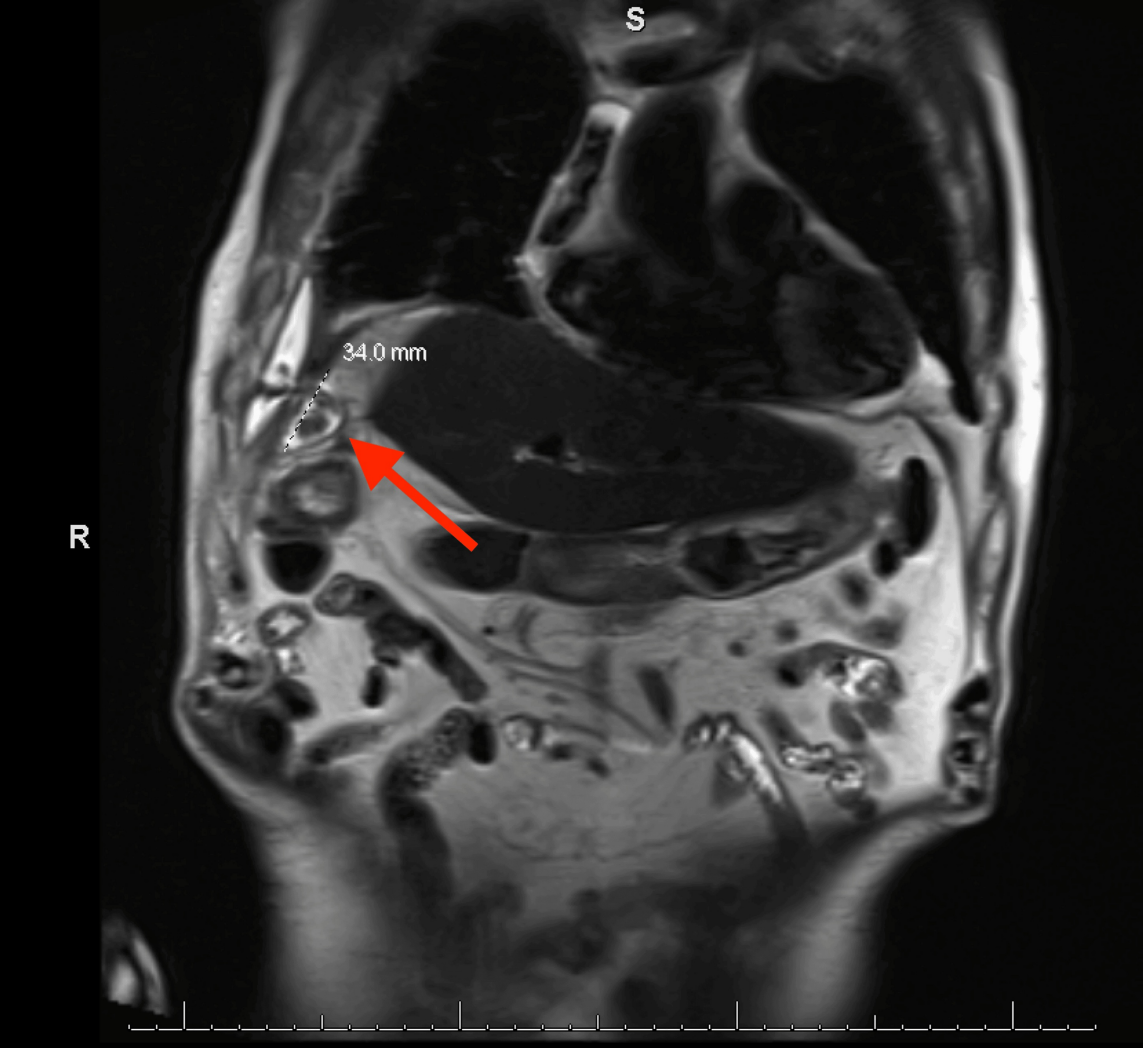
MRCP demonstrating findings consistent with gallbladder perforation and abscess formation in the right subphrenic region MRCP: magnetic resonance cholangiopancreatography

The right upper quadrant ultrasound revealed gallstones with concern for a ruptured gallbladder with a small amount of adjacent loculated fluid in the upper abdomen, while repeat CT with IV contrast reinforced prior findings of possible perforated cholecystitis with loculated right-sided empyema. Magnetic resonance cholangiopancreatography (MRCP) showed additional findings consistent with perforation at the fundus of the gallbladder with an adjacent pericholecystic and right subphrenic abscess (2.9 x 5.3 x 3.4 cm), which appeared to be inseparable from the right hemidiaphragm (Figure 3). No biliary dilatation or choledocholithiasis was noted.

The patient was evaluated by thoracic surgery and underwent right-sided video-assisted thoracoscopic surgery (VATS) with conversion to a posterolateral thoracotomy and complete decortication of the right lung due to the complexity of the empyema. The fluid within the lung appeared bilious with loculated pus. The fluid was cultured and sent to pathology for evaluation. The results were positive for Klebsiella aerogenes, which prompted the discontinuation of piperacillin/tazobactam and the initiation of cefepime, given the bacterial organism’s susceptibility.

**Figure 4:**
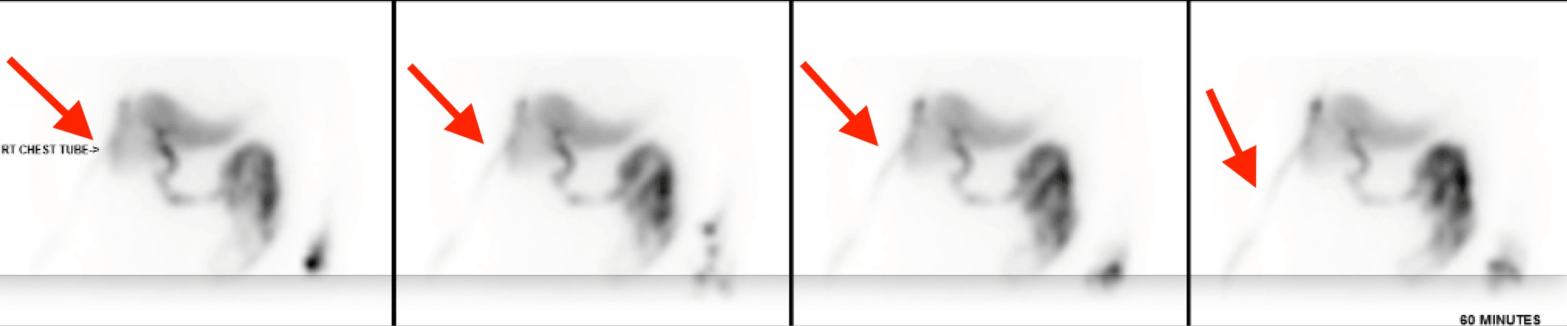
PIPIDA scan showing an abnormal accumulation of radiotracer above the right hepatic lobe and within the right-sided chest tube PIPIDA: 99mTc-paraisopropyliminodiacetic acid

Following VATS (postoperative day one), he underwent 99mTc-paraisopropyliminodiacetic acid (PIPIDA) cholescintigraphy for further evaluation of a possible gallbladder leak and the need for surgical intervention. The PIPIDA scan revealed an abnormal accumulation of radiotracer above the right hepatic lobe with significant radiotracer activity within the right-sided chest tube (Figure 4). These findings reinforced previous studies and were consistent with perforated gallbladder with communication with the right pleura.

After PIPIDA results were obtained, the patient was taken to the operating room by general surgery for robotic cholecystectomy, BPF takedown, and hepatic abscess drainage. During the procedure, severe inflammation of the transverse colon was noted, and leakage of bile was visualized, stemming from a friable portion of the diaphragm. No colonic interventions were performed. Firefly fluorescence imaging was utilized to properly identify the fistula, and the area was thoroughly irrigated for improved visualization prior to takedown. Next, the gallbladder was located, and biopsies were retrieved from both the gallbladder and liver abscess. Cholecystectomy was performed, and firefly fluorescence showed no biliary leakage. A 15 round abdominal Jackson-Pratt (JP) drain was placed intra-operatively to manage fluid collection and promote recovery. Pathology results from biopsies revealed acute and chronic inflammation within the liver abscess sample and chronic cholecystitis for the gallbladder sample.

The patient’s postoperative course was unremarkable. He was cleared by thoracic surgery on VATS postoperative day four. His abdominal JP drain was removed on robotic BPF takedown and cholecystectomy on postoperative day three when output was noted to be 5 mL in 24 hours. He was discharged home on postoperative day six. At that time, cefepime was discontinued, and he was started on a three-week course of levofloxacin 750 mg daily. He has followed up in the clinic and continues to do well postoperatively.

## Discussion

Acute cholecystitis, or inflammation of the gallbladder, is most commonly due to the obstruction of the cystic duct by gallstones and is generally treated via cholecystectomy. A delay in the diagnosis and treatment of cholecystitis can lead to severe complications, including perforation, empyema, gangrene, and sepsis [1,2]. Additionally, this delay in management can create a hostile environment for surgery, leading to surgical complications, including bowel and bile duct injuries. Gallbladder perforations may potentially cause fistula formation, adhesions with abscess formation, or bile efflux with cholangitis [2].

Cholelithiasis is the main cause of chronic gallbladder perforation [1]. Perforation occurs in roughly 5% of acute cholecystitis cases, and the incidence increases with age [3]. Older patients may not present with classical symptoms of cholecystitis, thereby delaying diagnosis and care. This stresses the importance of a high index of suspicion and noninvasive diagnostic testing to make a timely diagnosis. First-line imaging for the suspicion of cholecystitis is the ultrasound, which holds a sensitivity of 96%. MRCP, which has better diagnostic accuracy, allows physicians to analyze the biliary anatomy better [4].

BPF, a rare complication of cholelithiasis with subsequent perforation, may result from erosion of the diaphragm due to the corrosive nature of bile [1,5]. Invasion of the diaphragm creates pathologic communication with the pleural space, allowing bile’s irritating effects to produce respiratory and systemic symptoms, including shortness of breath, chest tightness, fever, and sepsis. BPFs may result from thoracoabdominal trauma, iatrogenic injury (following cholecystectomy, liver biopsy, etc.), inflammatory or neoplastic liver conditions, and infectious mechanisms such as parasitic liver disease [3,6,7]. BPFs related to biliary lithiasis are termed “primary fistulas” [4].

When a BPF is suspected and basic diagnostic tests (ultrasound and CT) are completed, more advanced modalities may be utilized to confirm the diagnosis and better evaluate the anatomy [1,5]. Additional biliary-focused diagnostic methods will allow for the determination of more precise fistula locations. Endoscopic retrograde cholangiopancreatography (ERCP) is a widely used study that is both diagnostic and therapeutic for patients [1,5]. MRCP and PIPIDA scans are used as well, to assess the location of the fistula. Understanding the cause of the fistula, as well as the level of the obstruction within the biliary tree and the location of the diaphragm injury, is essential in planning a successful surgical intervention [4]. Both MRCP and PIPIDA scans were used for our patient, which allowed for the confirmation of gallbladder perforation with right pleural communication. Thoracentesis is commonly used to reveal bile within the pleural fluid. Analysis can be performed to compare the total bilirubin level within the pleural fluid to that of serum, with a ratio over 3.0 being diagnostic [5,6,7]. Using the correct diagnostic tests when suspecting a biliary pleural fistula is crucial, as early diagnosis can reduce the rate of complications.

The treatment of biliary pleural fistulas is decided on a case-by-case basis. A review of the literature suggests ERCP with sphincterotomy as the first-line treatment [1,5,7]. If ERCP is not feasible, given difficult anatomy, chronic infection, or other contraindications, or if it fails to fully correct the fistula, surgical correction is indicated. The intervention typically involves cholecystectomy, which may be performed open or laparoscopically, depending on the severity of the obstruction [4,5]. The use of indocyanine green and robotic surgery may decrease the risk of complications in hostile anatomy. In addition to cholecystectomy, the procedure requires resection of the fistula, removal of any noted abscesses, and repair of the diaphragm [4].

## Conclusions

With this case, we hope to raise awareness when examining patients with pleural effusions or empyema, especially in those with concurrent gastrointestinal symptoms. Biliary pleural fistulas are very rare entities but keeping them in mind when constructing differential diagnoses can be key. We described a unique case and detailed the diagnostic workup and important findings, as well as the treatment approach taken by infectious disease, thoracic surgery, and general surgery. PIPIDA imaging was a vital diagnostic step used to pinpoint the precise location of the fistula. Additionally, the use of firefly fluorescence was significant in this case to effectively locate and treat the fistula and prevent sequential bile leakage. Treatment of BFs must be precise and prompt, as any delay increases the risk of serious complications.
